# Sinapic Acid Attenuated Cardiac Remodeling After Myocardial Infarction by Promoting Macrophage M2 Polarization Through the PPARγ Pathway

**DOI:** 10.3389/fcvm.2022.915903

**Published:** 2022-07-11

**Authors:** Mei Yang, Jun Xiong, Qiang Zou, Xi Wang, Ke Hu, Qingyan Zhao

**Affiliations:** ^1^Department of Cardiology, Renmin Hospital of Wuhan University, Wuhan, China; ^2^Cardiovascular Research Institute, Wuhan University, Wuhan, China; ^3^Hubei Key Laboratory of Cardiology, Wuhan, China; ^4^Department of Emergency, Renmin Hospital of Wuhan University, Wuhan, China; ^5^Department of Respiratory and Critical Care Medicine, Renmin Hospital of Wuhan University, Wuhan, China

**Keywords:** sinapic acid, myocardial infarction, macrophage, interstitial fibrosis, PPARγ

## Abstract

**Background:**

Macrophage polarization is an important regulatory mechanism of ventricular remodeling. Studies have shown that sinapic acid (SA) exerts an anti-inflammatory effect. However, the effect of SA on macrophages is still unclear.

**Objectives:**

The purpose of the study was to investigate the role of SA in macrophage polarization and ventricular remodeling after myocardial infarction (MI).

**Methods:**

An MI model was established by ligating the left coronary artery. The rats with MI were treated with SA for 1 or 4 weeks after MI. The effect of SA on bone marrow-derived macrophages (BMDMs) was also observed *in vitro*.

**Results:**

Cardiac systolic dysfunction was significantly improved after SA treatment. SA reduced MCP-1 and CCR2 expression and macrophage infiltration. SA decreased the levels of the inflammatory factors TNF-α, IL-1α, IL-1β, and iNOS and increased the levels of the M2 macrophage markers CD206, Arg-1, IL-10, Ym-1, Fizz-1, and TGF-β at 1 week after MI. SA significantly increased CD68^+^/CD206^+^ macrophage infiltration. Myocardial interstitial fibrosis and MMP-2 and MMP-9 levels were decreased, and the sympathetic nerve marker TH and nerve sprouting marker GAP43 were suppressed after SA treatment at 4 weeks after MI. The PPARγ level was notably upregulated after SA treatment. *In vitro*, SA also increased the expression of PPARγ mRNA in BMDMs and IL-4-treated BMDMs in a concentration-dependent manner. SA enhanced Arg1 and IL-10 expression in BMDMs, and the PPARγ antagonist GW9662 attenuated M2 macrophage marker expression.

**Conclusions:**

Our results demonstrated that SA attenuated structural and neural remodeling by promoting macrophage M2 polarization *via* PPARγ activation after MI.

## Introduction

Monocytes are recruited to the necrotic and ischemic myocardium and then become plastic macrophages after myocardial infarction (MI). For a few days in the early stage of MI, polarized M1 macrophages secrete proinflammatory mediators to activate an intense inflammatory cascade response, and polarized M2 macrophages persist for weeks to participate in fibrosis and remodeling (1). Interstitial fibrosis after MI is the main cause of heart failure, and sympathetic neural remodeling contributes to ventricular arrhythmia that is closely associated with sudden cardiac death (2, 3). Studies have shown that modulation of macrophage polarization is a potential therapeutic target for cardiac remodeling after MI (4).

Previous studies have shown that ligand-dependent nuclear receptor peroxisome proliferator-activated receptor γ (PPARγ) can regulate the phenotype of macrophages and participate in M2 macrophage polarization (5, 6). Experiment research showed that PPARγ is expressed in macrophages and that PPARγ ligands can induce the expression of M2 macrophage markers *in vitro* (7).

Sinapic acid (SA) is a hydroxycinnamic acid derivative that has been proven to have protective effects on different disease models, such as liver injury (8), hepatitis (9), nephrotoxicity (10, 11), gastric ulcer (12), lung fibrosis (13) and epilepsy (14). An increasing number of studies have also found that SA exerts a cardioprotective effect. SA has antilipidemic and antioxidant effects in the early stage of myocardial damage (15, 16). SA protects against hypertension by modulating reactive oxygen species and inhibiting fibrosis (17, 18). In addition, studies found that SA could ameliorate acute DOX-induced cardiotoxicity by inhibiting inflammation (19), and protect against streptozotocin (STZ)-induced diabetic cardiac dysfunction (20). Recently, one study has shown that SA attenuates cisplatin-induced nephrotoxicity by PPARγ activation (21). However, to the best of our knowledge, whether SA inhibits development of ventricular remodeling after acute MI has not been investigated. The purpose of this study was to test the hypothesis that SA attenuated cardiac remodeling after acute MI by promoting macrophage M2 polarization through the PPARγ pathway.

## Materials and Methods

### Animals and Experimental Group

All animal procedures were performed in agreement with the Animal Care and Use Committee of Renmin Hospital of Wuhan University (Wuhan, China) and complied with the guidelines of the National Institutes of Health for the care and use of laboratory animals. Sprague Dawley rats (male, 6–8 weeks) were randomly divided into the sham group (*n* = 10), MI group (*n* = 10), and MI+SA group (*n* = 10). In the MI+SA group, rats were treated with SA (Med Chem Express, USA) (20 mg/kg/d) by intragastric administration 24 h after acute MI for 1 or 4 weeks. SA was dissolved in corn oil (5 mg/mL). The rats in the MI and sham groups were administered corn oil by intragastric administration.

### Acute MI Model Establishment

Rats were anesthetized with an intraperitoneal injection of sodium pentobarbital (50 mg/kg) as previous study (22). The rats then underwent endotracheal intubation and surface electrocardiography. A thoracotomy was performed, and the heart was exposed. Left anterior descending coronary artery ligation was performed by a 6-0 non-invasive suture according to our previous method (23). In the sham group, the suture was only crossed under the left anterior descending coronary artery without ligation.

### Cell Culture and Treatment

Bone marrow was isolated from the femurs and tibias of rats according to our previous method (24). Red blood cell lysate was added for 5 min and then washed with PBS. Cells were cultured in Dulbecco's modified Eagle's medium with 10% fetal bovine serum and 30 μg/L macrophage colony-stimulating factor. Following 48 h of culture in an incubator at 37°C with a 5% CO_2_ atmosphere, the medium was removed and replaced with fresh medium every 3 days. Bone marrow-derived macrophages (BMDMs) were randomly divided into six groups to observe the effect of SA on M2 polarization: a control group, IL-4 group, SA group, IL-4+SA group, SA+GW9662 group, and IL-4+SA+GW9662 group. BMDMs in the LPS group and IL-4 group were treated with LPS (100 ng/mL) or IL-4 (10 ng/mL) for 24 h. SA (100 or 200 μmol/L) was simultaneously added for 24 h. In the SA+GW9662 and IL-4+SA+GW9662 groups, BMDMs were pretreated with the PPARγ antagonist GW9662 (5 μmol/L) (HY-16578, Med Chem Express, USA).

### Echocardiography

Echocardiography was used to evaluate left ventricular (LV) systolic function after 1 and 4 weeks. Echocardiography was recorded at the papillary muscle level in the LV short axis. The transducer frequency was 10 MHz. The parameters included LV end-diastolic dimension (LVEDD) and LV ejection fraction (LVEF).

### Western Blot Analysis

Total protein was extracted from myocardial tissue in the peripheral zone of MI and BMDMs, and the protein concentration was measured by a bicinchoninic acid assay kit. The sample loading amount was determined according to the sample concentration to ensure that the total protein loading amount of each sample was 40 μg. Sodium dodecyl sulfate–polyacrylamide gel electrophoresis was performed, and the proteins were transferred to a polyvinylidene difluoride membrane. The membrane was incubated with primary antibodies against MCP-1 (Bioss, bs-34021R, 1:500), CCR2 (Biorbyt, orb378630, 1:1,000), Arg-1 (Santa Cruz, sc-271430, 1:500), MMP-2 (Abcam, ab92536, 1:1,000), MMP-9 (Abcam, ab76003, 1:1,000), and PPARγ (Abcam, ab209350, 1:1,000) overnight at 4°C and then incubated with HRP-conjugated goat anti-rabbit secondary antibody (ASPEN, AS1107, 1:10,000) at 37°C for 45 min; then, enhanced chemiluminescence detection was performed. Optical density was detected by the AlphaEaseFC software system.

### Reverse Transcription-Quantitative Polymerase Chain Reaction

Total RNA was extracted from the peripheral area of infarction by using TRIzol® reagent. The UV spectrophotometer measures the A260 and A280 values to detect the purity of RNA and ensure that the A260/A280 ratio is between 2.0 and 2.2. The isolated RNA was converted into cDNA using the PrimeScript™ RT reagent kit with gDNA Eraser (TaKaRa, RR047A). The primers were synthesized by Invitrogen Biotechnology (Shanghai, China) and are presented in Table 1. RT–qPCR was performed using the StepOne™ Real-Time PCR system (Life Technologies, Carlsbad, CA, USA). The reactions were then conducted using SYBR® Premix Ex Taq TM II (Takara Bio, Japan, RR420A). Semilog amplification curves were analyzed using the 2-ΔΔCt comparative quantification method (25), and the expression of each gene was normalized to that of GAPDH.

**Table 1:** PCR primers used in this study.

**Gene**	**Primer**	**Product size (bp)**
TNF-α	Sense:5′- CACCACGCTCTTCTGTCTACTG - 3′	147
	Antisense:5′- GCTACGGGCTTGTCACTCG - 3′	
IL-1α	Sense:5′- GATCAGCACCTCACAGCTTCC - 3′	204
	Antisense:5′- TAGAGTCGTCTCCTCCCGATG - 3′	
IL-1β	Sense:5′- GTGGCAGCTACCTATGTCTTGC - 3′	251
	Antisense:5′- CCACTTGTTGGCTTATGTTCTGT - 3′	
IL-10	Sense:5′- ACTGCTATGTTGCCTGCTCTTAC - 3′	199
	Antisense:5′- CAGTAAGGAATCTGTCAGCAGTATG - 3′	
NGF	Sense:5′- GATAAGACCACAGCCACGGAC - 3′	182
	Antisense:5′- TGAGTCGTGGTGCAGTATGAGTT - 3′	
PPARγ	Sense:5′- CCTTTACCACGGTTGATTTCTC - 3′	141
	Antisense:5′- CAGGCTCTACTTTGATCGCACT - 3′	
Arg-1	Sense:5′- ATTGGCAAAGTGATGGAAGAGAC - 3′	287
	Antisense:5′- CAAGACAAGGTCAACGCCAC - 3′	
iNOS	Sense:5′- AGCATCCACGCCAAGAACG - 3′	167
	Antisense:5′- GTCTGGTTGCCTGGGAAAAT - 3′	
Ym-1	Sense:5′- GTTTCAAAATTGGTAATATTGACCC - 3′	159
	Antisense:5′- GAGTTTTTAGCTCAGTGTTCCTGTC - 3′	
Fizz-1	Sense:5′- TAGACATTATTGGGAAGAAAAAGGT - 3′	202
	Antisense:5′- TTGCAAATATTTTCATTCTGGATTT - 3′	
TGF-β	Sense:5′- AAGGAGACGGAATACAGGGCT - 3′	107
	Antisense:5′- ACCTCGACGTTTGGGACTGA - 3′	
GAPDH	Sense:5′- GCCAAGGTCATCCATGACAAC - 3′	152
	Antisense:5′- GTGGATGCAGGGATGATGTTC - 3′	

### Masson Trichrome Staining

The myocardial tissue in the peripheral infarct zones was fixed with 4% paraformaldehyde and embedded in paraffin. The sections were dewaxed, dehydrated, and stained with hematoxylin. Next, the sections were stained with Ponceau and aniline blue after washing and sealed with neutral gum. The collagen volume fraction was defined as the ratio of the collagen fiber area to the view area.

### Immunofluorescence

The paraffin sections were dewaxed and hydrated and then blocked with 5% bovine serum albumin for 20 min. Next, the sections were incubated with the primary antibodies CD68 (Abcam, ab125212; 1:200) and CD206 (Abcam, ab64693; 1:200) (Monoclonal antibody, 60143-1-Ig, 1:10,000) overnight at 4°C and the secondary antibody Cy3-conjugated AffiniPure goat anti-rabbit IgG (Aspen, AS1109, 1:50) at 37°C for 50 min. 4′,6-Diamidino-2-phenylindole (DAPI) was used to visualize the nuclei. The sections were observed by the ScanScope system. Four visual fields were randomly selected in every section. The CD68+ and CD206+ counts were the number of CD68-positive cells and CD206-positive cells per field.

### Immunohistochemistry

The primary antibodies TH (Abcam, ab109189, 1:200) and GAP43 (Abcam, ab128005, 1:150) were added to the sections overnight at 4°C. Then, the sections were incubated with horseradish peroxidase-conjugated goat anti-rabbit IgG (Aspen, AS1107, 1:200) secondary antibody at 37°C for 50 min. Then, the sections were stained with diaminobenzidine solution. Four visual fields were randomly selected in every section. The mean integral optical density of TH and GAP43 was measured by Image-Pro Plus software.

### Statistical Analysis

The data are expressed as the mean ± standard deviation. The statistical significance of the differences among groups was determined using one-way analysis of variance with Tukey's *post-hoc* test. Comparisons between two groups were made using the unpaired and two-tailed *t*-test. The sample number of experiments was set before data were obtained. Experimental replicates were not used to increase the *n*-value for statistical purposes. *P* < 0.05 was considered to indicate a statistically significant difference.

## Results

### SA Ameliorated Cardiac Systolic Dysfunction

**Figure 1:**
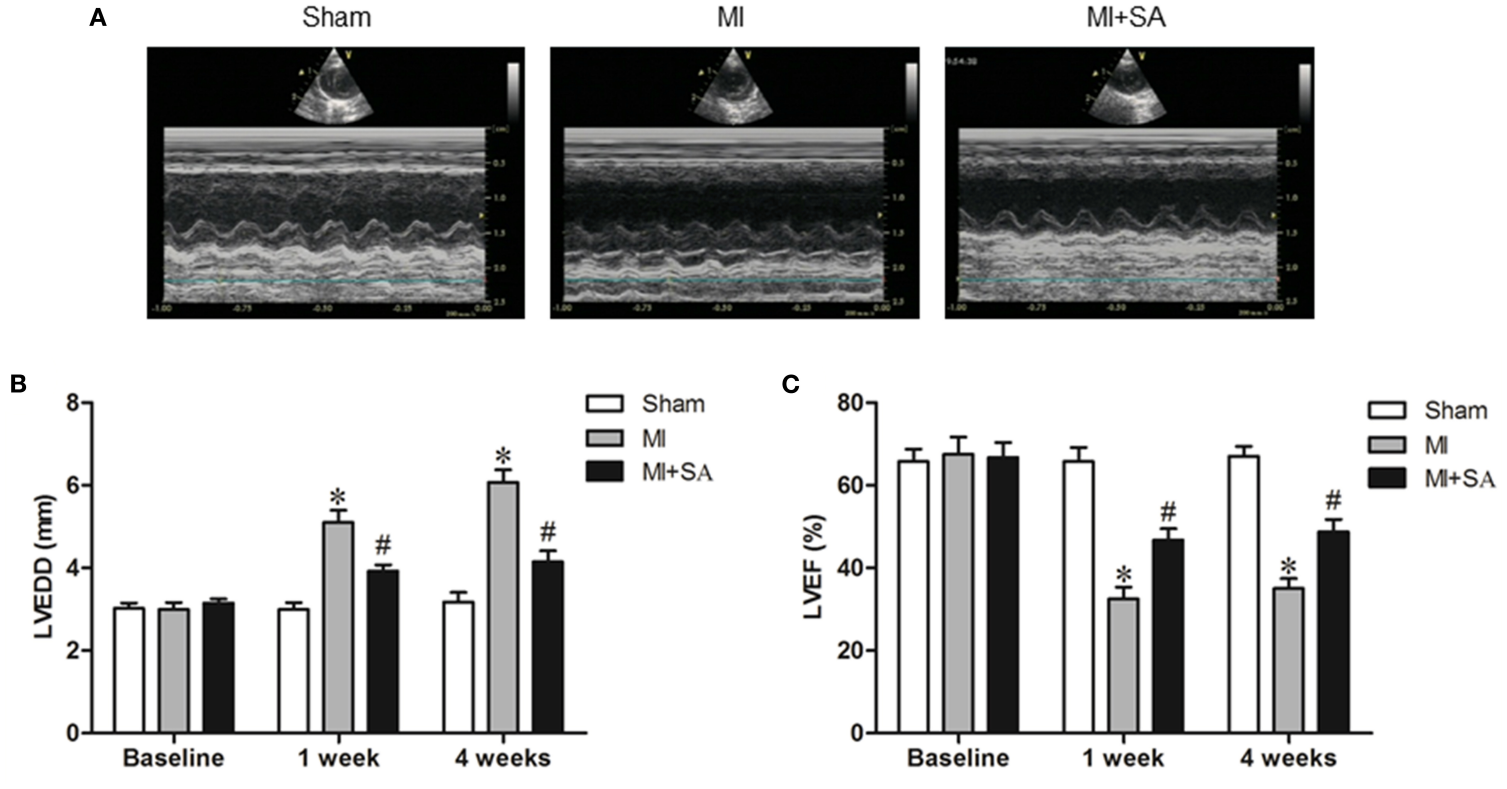
SA ameliorated cardiac dysfunction after MI. **(A)** Echocardiographic images in the three groups at 1 week after MI. **(B,C)** SA decreased LVEDD and increased LVEF at 1 week and 4 weeks after MI. ^*^Compared with the sham group, *P* < 0.05, ^#^Compared with the MI group, *P* < 0.05, *n* = 8 in each group.

In the MI group, LVEDD increased and LVEF decreased compared with the sham group after 1 and 4 weeks (Figure 1). After SA treatment, LVEDD was reduced and LVEF was improved compared with the MI group. SA significantly ameliorated LV dysfunction after MI (Figure 1). There was no significant difference in LVEDD or LVEF between 1 and 4 weeks after myocardial infarction in the three groups.

### SA Reduced Macrophage Infiltration and Promoted Macrophage M2 Polarization

**Figure 2:**
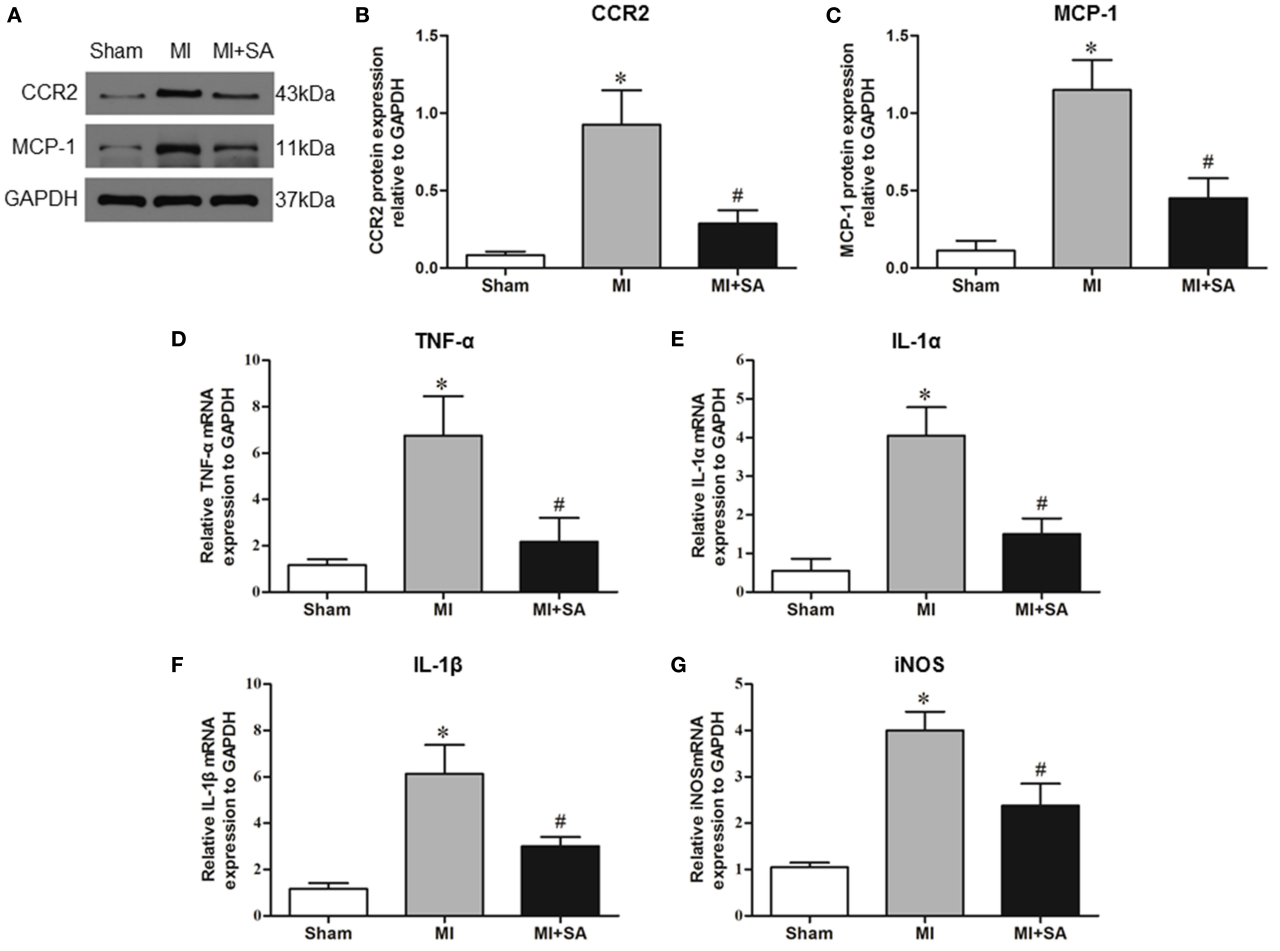
SA inhibited inflammatory factors at 1 week after MI. **(A–C)** MCP-1 and CCR2 in the three groups were detected by Western blot. **(D–G)** Levels of the inflammatory factors TNF-α, IL-1α, IL-1β, and iNOS were detected by RT–qPCR. *Compared with the sham group, *P* < 0.05, ^#^Compared with the MI group, *P* < 0.05, *n* = 8 in each group.

**Figure 3:**
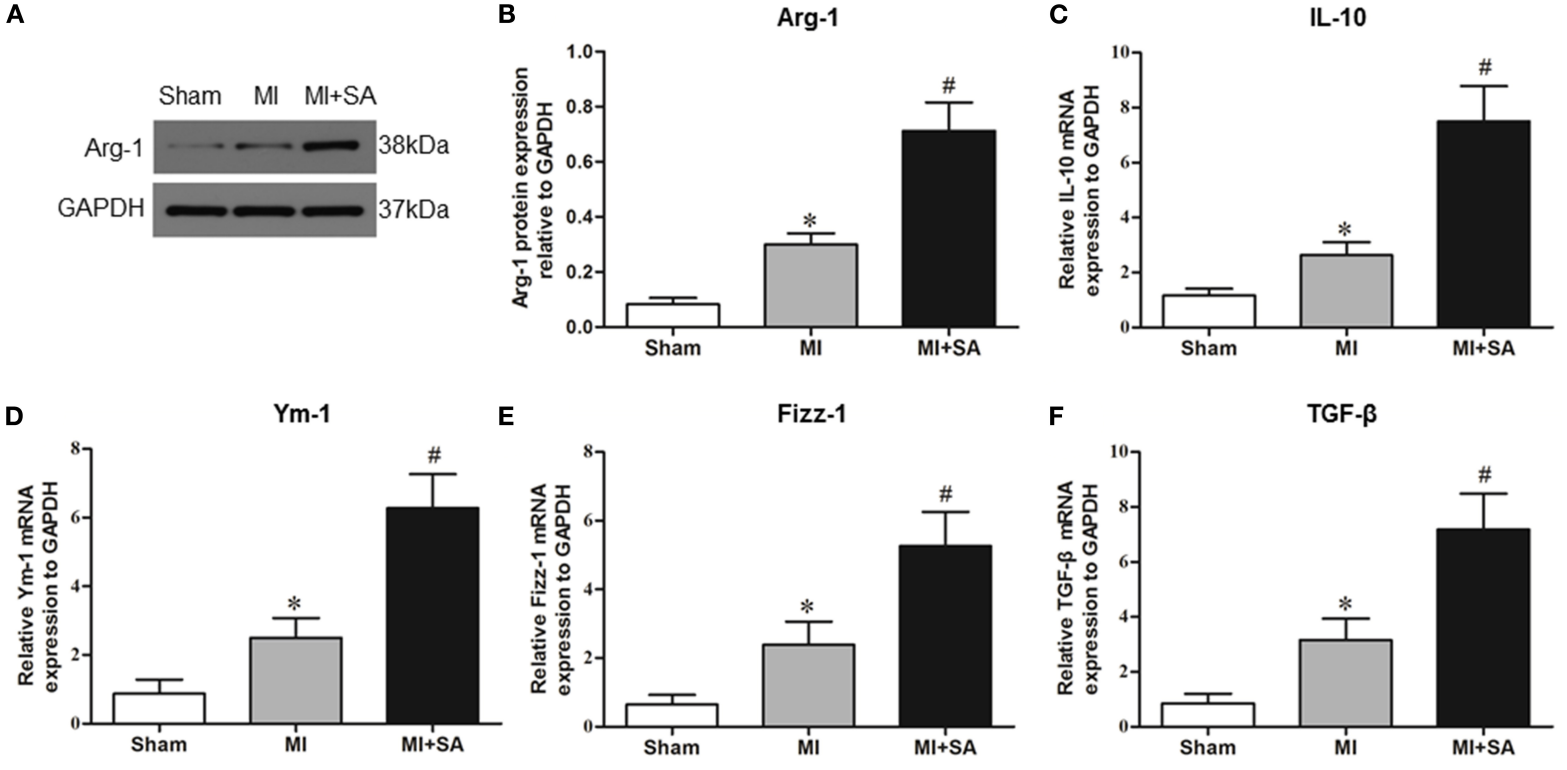
SA promotes M2 macrophage marker expression at 1 week after MI. **(A,B)** Arg-1 in the three groups was detected by Western blot. **(C–F)** Levels of the M2 macrophage markers IL-10, Ym-1, Fizz-1, and TNF-β were detected by RT–qPCR. *Compared with the sham group, *P* < 0.05, ^#^Compared with the MI group, *P* < 0.05, *n* = 8 in each group.

**Figure 4:**
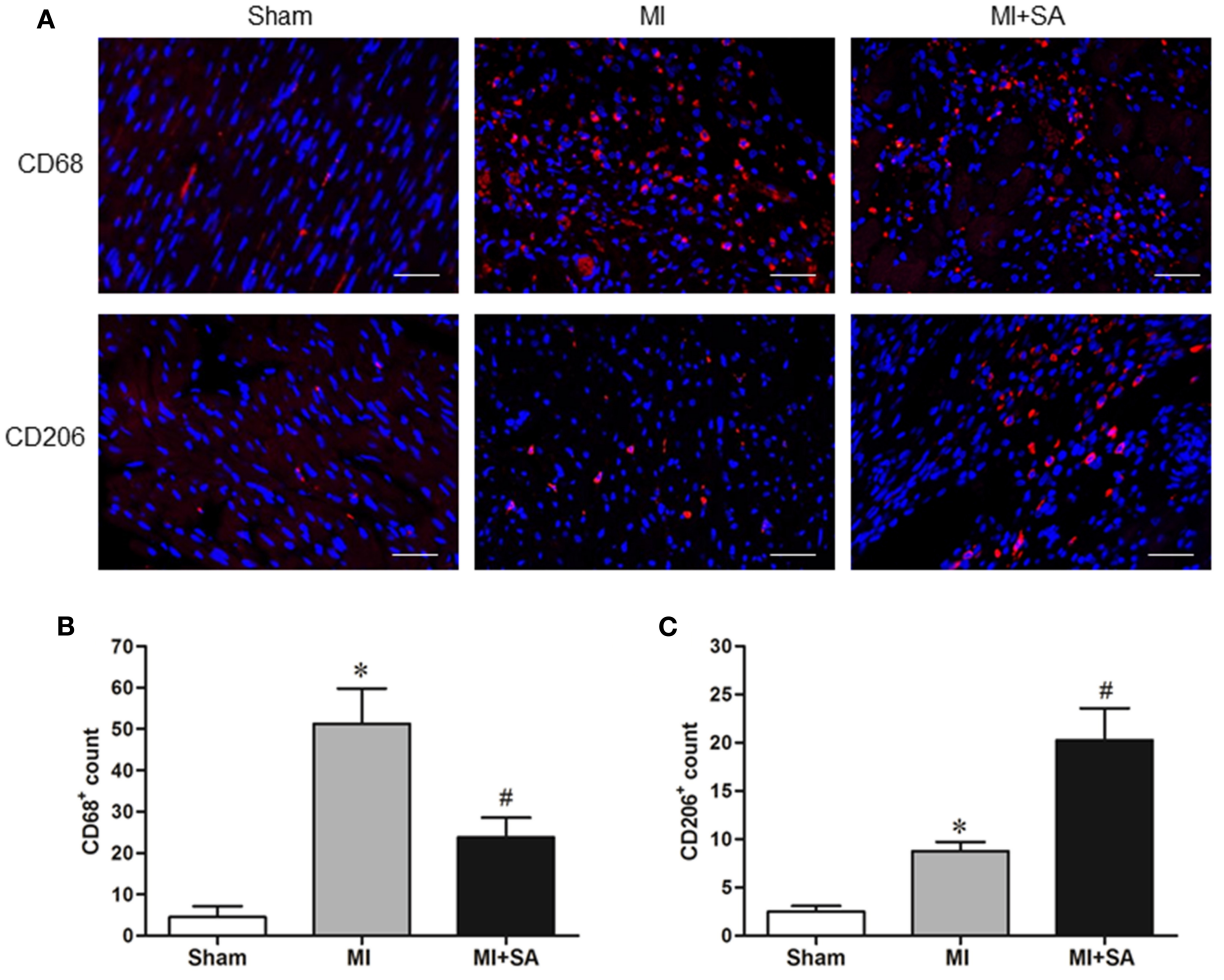
SA decreased CD68-positive macrophage infiltration and induced M2 macrophage polarization at 1 week after MI. **(A)** The general macrophage marker CD68 and the M2 macrophage marker CD206 were detected by immunostaining analysis. Positive staining of CD68 and CD206 is red. The nuclei were counterstained with DAPI (blue). Scale bar, 100 μm. **(B,C)** Immunostaining quantitative analysis showed that CD68 expression was decreased and CD206 expression was increased after SA treatment. *Compared with the sham group, *P* < 0.05, ^#^Compared with the MI group, *P* < 0.05, *n* = 8 in each group.

**Figure 5:**
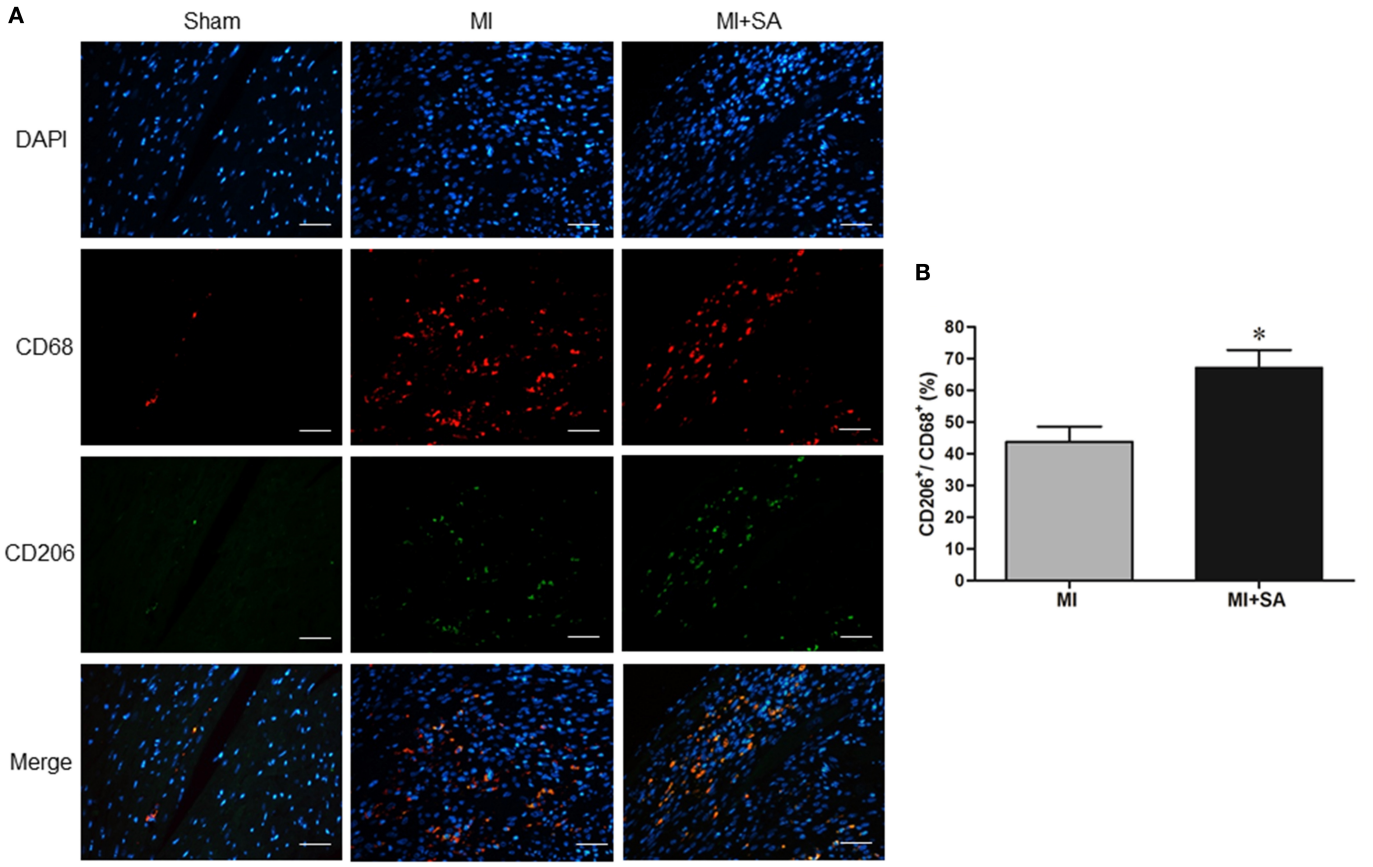
SA increased CD68^+^/CD206^+^ macrophage infiltration at 1 week after MI. **(A)** CD68^+^ and CD206^+^ positive cells detected by immunostaining analysis. Positive staining of CD68 is red, and CD206 is green. The nuclei were counterstained with DAPI (blue). Scale bar, 100 μm. **(B)** Immunostaining quantitative analysis showed that CD68+/CD206+ macrophages were increased after SA treatment. *Compared with the sham group, *P* < 0.05, *n* = 8 in each group.

The effect of SA on macrophages was analyzed at 1 week after MI. SA markedly reduced the increased MCP-1 and CCR2 protein levels after MI (Figures 2A–C). The inflammatory factors TNF-α, IL-1α, IL-1β, and iNOS were decreased in the MI+SA group compared with the MI group (Figures 2D–G). The effect of SA on macrophage polarization was further analyzed, and our study found that SA significantly enhanced the levels of the M2 markers Arg-1, IL-10, Ym-1, Fizz-1, and TGF-β (Figures 3A–F). Immunofluorescence analysis showed that the expression levels of the general macrophage marker CD68 and the M2 macrophage marker CD206 were both upregulated in the MI group compared with the sham group. CD68-positive cell infiltration was greatly decreased, and CD206-positive cell infiltration was greatly increased following SA treatment at 1 week after MI (Figures 4A–C). Further study found that the percentage of CD206/CD68 double-labeled cells notably increased (43.75 ± 4.79% vs. 67.0 ± 5.72%) (Figures 5A,B), suggesting that SA promotes macrophage M2 polarization.

### SA Attenuated Myocardial Fibrosis

**Figure 6:**
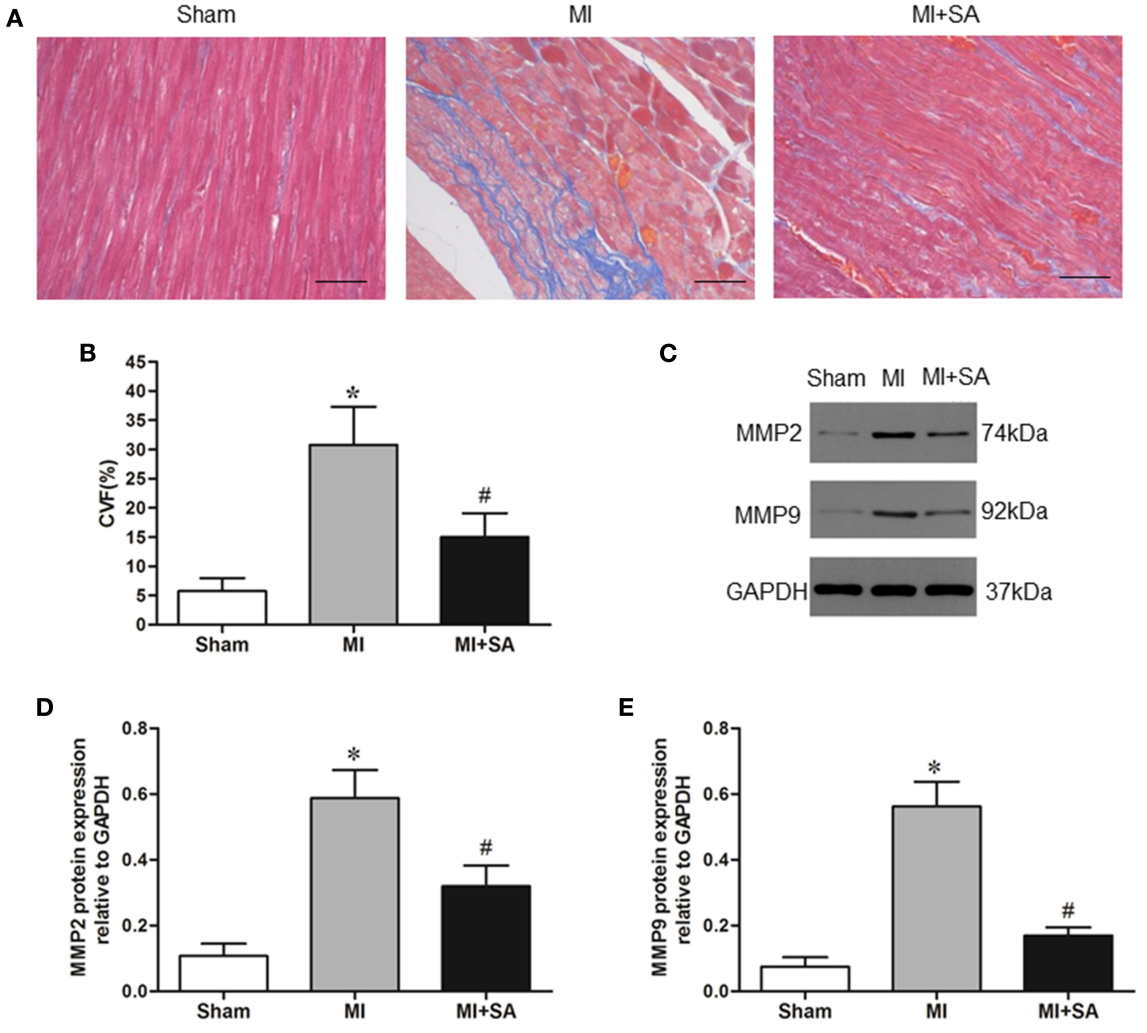
The effect of SA on myocardial fibrosis at 4 weeks after MI. **(A)** Interstitial fibrosis (blue) detected by Masson staining in the sham group, MI group, and MI+SA group. Scale bar, 50 μm. **(B)** Comparison of collagen volume fraction. **(C)** Expression of MMP9 and MMP2 proteins in the three groups. **(D,E)** Quantitative assessment of MMP9 and MMP2 protein levels. *Compared with the sham group, *P* < 0.05, ^#^Compared with the MI group, *P* < 0.05, *n* = 8 in each group.

Myocardial fibrosis was evaluated by collagen synthesis and collagen degradation. Masson staining and quantitative analysis of the collagen volume fraction indicated that SA significantly attenuated interstitial fibrosis after 4 weeks of acute MI (Figures 6A,B). MMP-2 and MMP-9 protein levels were also elevated after MI and markedly reduced after SA treatment (Figures 6C–E).

### Effect of SA on Neural Remodeling

**Figure 7:**
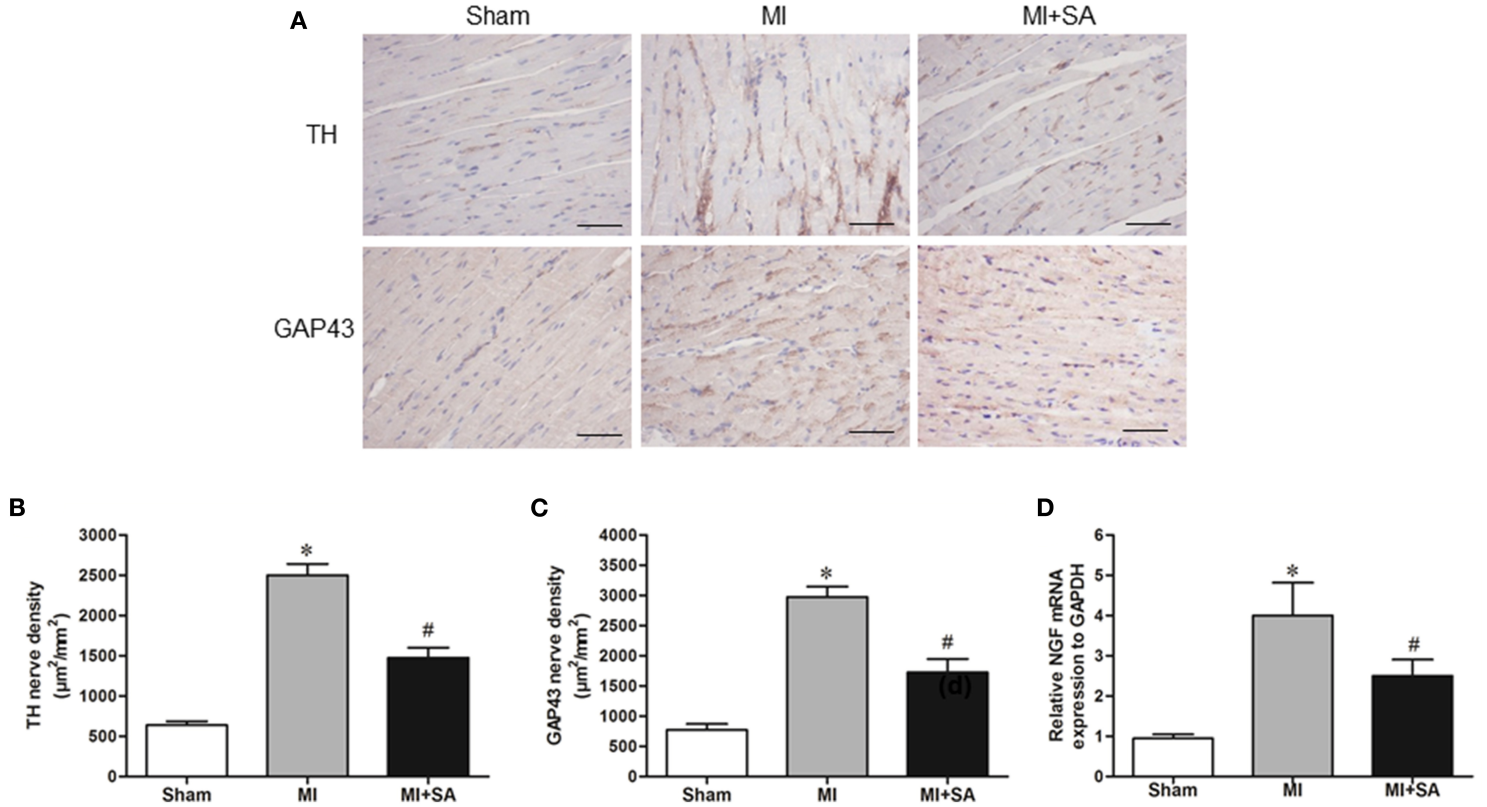
SA attenuated sympathetic nerve remodeling and nerve sprouting at 4 weeks after MI. **(A)** TH and GAP43 were detected by immunohistochemistry. Scale bar, 50 μm. **(B,C)** Quantitative analysis of TH and GAP43. **(D)** Expression of NGF mRNA in the three groups. *Compared with the sham group, *P* < 0.05, ^#^Compared with the MI group, *P* < 0.05, *n* = 8 in each group.

The sympathetic nerve marker TH and the nerve sprouting marker GAP43 were evaluated by immunohistochemistry. There were a small number of TH-positive and GAP43-positive nerves in the sham group (Figure 7A). At 4 weeks after MI, cardiac sympathetic nerve fibers and nerve sprouting were significantly increased in the peripheral area of MI (Figures 7A–C). Compared with the MI group, the results of nerve density measurements indicated that TH-positive and GAP43-positive nerves were notably lower in the MI+SA group (Figures 7A–C). Furthermore, SA inhibited NGF expression after MI (Figure 7D).

### Effect of SA on the Expression of PPARγ *in vivo* and Effect of SA on BMDMs

**Figure 8:**
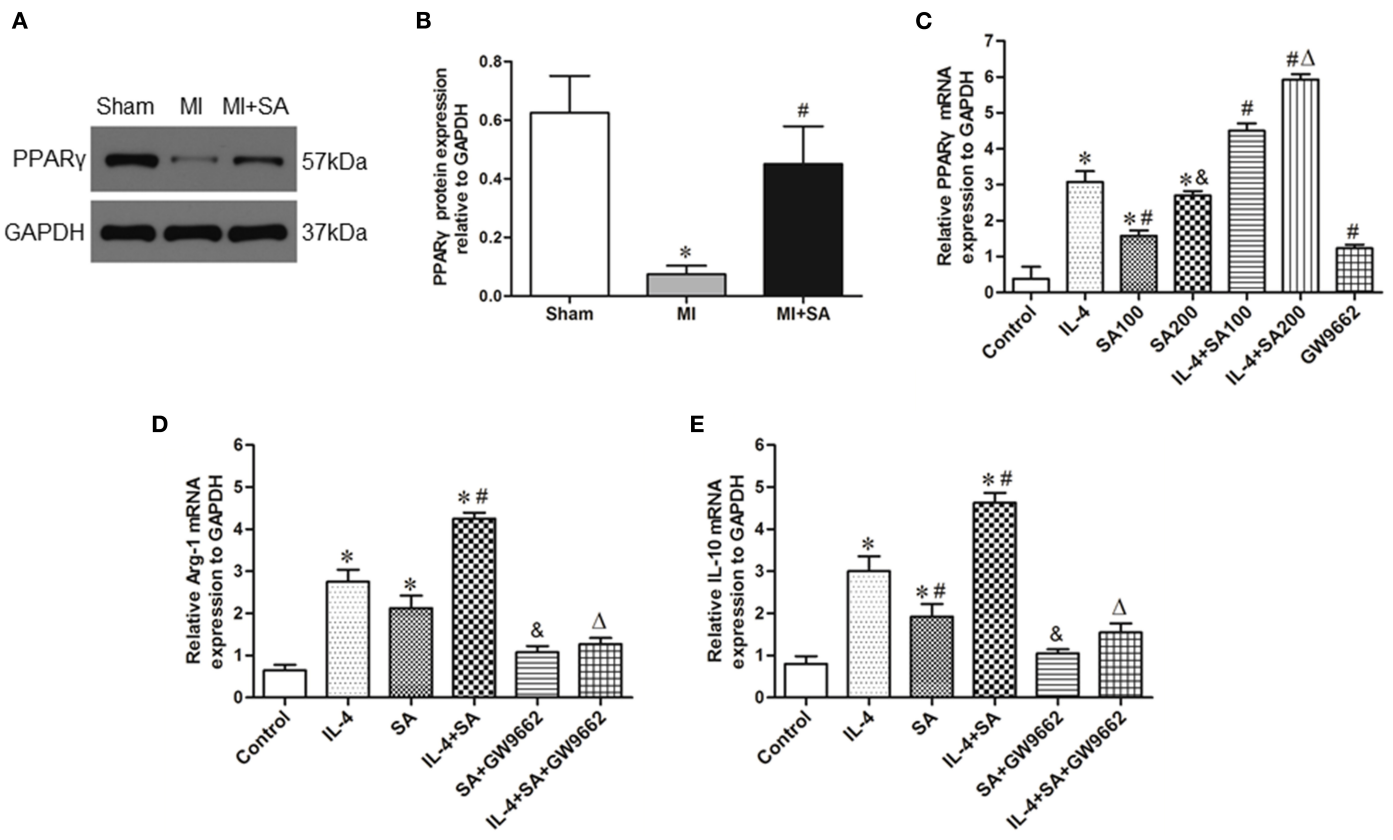
SA activated the expression of PPARγ and the effect of SA on BMDMs. **(A,B)** PPARγ protein expression was detected by Western blot analysis. *Compared with the sham group, *P* < 0.05, ^#^Compared with the MI group, *P* < 0.05. **(C)** PPARγ mRNA expression in BMDMs and IL-4-treated BMDMs after SA treatment. *Compared with the control group, *P* < 0.05; ^#^Compared with the IL-4 group, *P* < 0.05; ^&^Compared with the SA100 group, *P* < 0.05; ^Δ^Compared with the IL-4+SA100 group, *P* < 0.05. **(D,E)** The effect of SA on Arg1 and IL-10 mRNA expression in BMDMs and IL-4-treated BMDMs and the effect of the PPARγ antagonist GW9662 on SA-induced Arg1 and IL-10 expression. *Compared with the control group, *P* < 0.05; ^#^Compared with the IL-4 group, *P* < 0.05; ^&^Compared with the SA group, *P* < 0.05; ^Δ^Compared with the IL-4+SA group, *P* < 0.05. *n* = 6 in each group.

The expression of PPARγ protein was upregulated at 1 week after MI. In the MI+SA group, PPARγ expression was significantly elevated compared with that in the MI group (Figures 8A,B). To further investigate the mechanism of SA on macrophages, we observed the effect of SA on BMDMs *in vitro*. Our study found that SA also increased the expression of PPARγ mRNA in BMDMs and IL-4-treated BMDMs in a concentration-dependent manner (Figure 8C). SA and IL-4 both enhanced Arg1 and IL-10 expression, and there was no significant difference in Arg1 expression between the two groups. SA also elevated Arg1 and IL-10 levels in IL-4-treated BMDMs (Figures 8D,E). The PPARγ antagonist GW9662 significantly inhibited PPARγ mRNA level and attenuated SA- and IL-4+SA-induced M2 macrophage marker expression (Figures 8D,E).

## Discussion

This study explored the influence of SA on macrophage polarization and ventricular remodeling in a rat model of MI. We provide evidence for the following: (1) SA increased M2 macrophage infiltration in the peripheral infarct zones after MI; (2) SA attenuated myocardial interstitial fibrosis and neural remodeling after MI; (3) SA induced and promoted macrophage M2 polarization in BMDMs and IL-4-treated BMDMs *in vitro*; and (4) activation of PPARγ is a potential mechanism by which SA regulates macrophage polarization.

The plasticity of macrophages makes macrophages an important regulatory target for the treatment of myocardial infarction. After myocardial infarction, pro-inflammatory M1 macrophages aggravate inflammatory response and myocardial injury by releasing proinflammatory cytokines, exosomes and miRNA (26). An excessive inflammatory response and prolonged M1 macrophage accelerates myocardial injury and adverse cardiac remodeling (27, 28). In the present study, we also found that the inflammatory factors increased and macrophage infiltration in the peri-infarct area after MI. Interestingly, SA not only downregulated the inflammatory factors TNF-α, IL-1α, and IL-1β but also affected macrophage infiltration in the peri-infarct area after MI. Previous studies have shown that SA can inhibit the production of inflammatory factors after nephrotoxicity and hepatic injury (10, 29) and that SA represses TNF-α and IL-1β in DOX-induced cardiotoxicity (19). Other studies also found that MCP-1/CCR2 inhibition significantly ameliorated macrophage recruitment and interstitial fibrosis and improved heart function after ischemia and reperfusion injury and MI (30, 31). In addition, we found that SA reduced iNOS expression, which is an M1 macrophage marker, indicating that SA can inhibit the polarization of M1 macrophages after MI.

M1 and M2 macrophages are functionally heterogeneous. M2 macrophage infiltration begins to activate at 5–7 days after MI (32, 33). Immunofluorescence revealed that SA can increase CD68^+^/CD206^+^ macrophage infiltration by 23%, which suggested that SA can promote the polarization of M2 macrophages. M2 macrophages secrete various anti-inflammatory factors and many studies have shown that activation and increase of M2 macrophages can attenuate ventricular remodeling (34–37). Inhibition or depletion of M2 macrophage inhibiting M2 macrophage activationresulted in deterioration of cardiac function, enlargement of infarct area and increase of inflammatory cell infiltration after MI (38, 39). We further observed the effect of SA on IL-4-treated BMDMs. IL-4 was applied to modulate macrophage polarization toward an M2 phenotype. One study found that IL-4 administration can significantly increase M2 macrophage infiltration, reduce the MI area and improve cardiac function in mice with MI, and this effect depends on M2 macrophages rather than the direct effect of IL-4 (40). In both *in vivo* and *in vitro* experiments, SA promoted and enhanced the expression of M2 macrophage markers, such as Arg-1, IL-10, Ym-1, Fizz-1, and TGF-β, at 1 week after MI. We suggest that promoting M2 polarization is one of the new important anti-inflammatory mechanisms of SA.

The persistent inflammatory response recruits and activates myofibroblasts that synthesize extracellular matrix proteins, which are involved in myocardial remodeling. Previous studies have shown that SA prevents cardiac fibrosis in a hypertensive animal model (17). Our study demonstrated that not only cardiac fibrosis but also MMP-2 and MMP-9 expression was inhibited after SA treatment. MMP-2 and MMP-9 are key regulators of LV remodeling and were upregulated both in MI and heart failure (41). In MMP2 and MMP9 knockout mice, LV enlargement and collagen accumulation were significantly attenuated after MI (42, 43). Clinical studies have also shown that MMP-9 is an independent risk factor for heart failure after acute MI (44). Our study indicated that MMP2 and MMP9 are regulatory targets of the antifibrotic effect of SA. Interstitial fibrosis gradually leads to impaired cardiac function and eventually progresses to heart failure. Our study demonstrated that SA ameliorated ventricle dilatation and systolic dysfunction at 1 and 4 weeks after MI. These results suggested that SA may delay cardiac remodeling and heart failure by inhibiting inflammation and fibrosis.

Previous studies have shown that inhibition of the inflammatory response can improve sympathetic remodeling (45, 46). Furthermore, macrophage reduction followed by intravenous injection of clodronate inhibited sympathetic hyperinnervation after MI (47). A previous study showed that macrophages that synthesize and express NGF around sympathetic nerves participate in sympathetic sprouting after MI (48). In this study, we found that SA reduced sympathetic nerve density and nerve sprouting by inhibiting inflammation and macrophage infiltration. We also found that SA significantly inhibited the expression of NGF. Other studies have demonstrated the inflammatory factors TNF-α and IL-1β also directly regulate the expression of NGF (49). More importantly, M2 macrophages are closely related to nerve remodeling. NGF secretion decreased significantly after M2 polarization of microglia (50). One study showed that atorvastatin induced M2 macrophages and attenuated sympathetic hyperinnervation in rats post-myocardial infarction (51). Taken together, our findings suggest that SA may inhibit NGF expression and regulate nerve remodeling by promoting M2 macrophage polarization.

The other major findings of this study were that SA activated PPARγ in BMDMs in a concentration-dependent manner. PPARγ is closely associated with M2 polarization (5). Some drugs, such as rosuvastatin and pioglitazone, can improve M2 macrophage polarization by PPARγ activation (52, 53). IL-4 stimulation can induce BMDMs to M2 polarized activation and elevate PPARγ expression (54). In IL-4-stimulated PPARγ null BMDMs, the expression of the M2 macrophage marker Arg-1 was reduced by nearly half (6). More importantly, there is a PPAR response element (PPRE) upstream of the Arg-1, Ym-1, and Fizz-1 promoters to regulate the transcription of target genes (5, 6). One recent study found that SA possesses a PPARγ activation role and that the antioxidant stress effect of SA was abolished by the PPARγ inhibitor BADGE (21). In our study, we also found that SA activated PPARγ in BMDMs in a concentration-dependent manner. More importantly, in the present study, the PPARγ antagonist GW9662 attenuated IL-4-induced M2 macrophage marker expression after SA treatment, which indicated that PPARγ is the core signaling pathway by which SA regulates macrophage polarization. However, the specific mechanism of SA-mediated PPARγ activation remains to be further studied.

In conclusion, SA alleviated inflammation by promoting M2 macrophage polarization by activating the PPARγ pathway, and SA attenuated structural and neural remodeling by inhibiting inflammation. SA could be a therapeutic candidate for anti-inflammation and ventricular remodeling after MI.

### Limitations and Future Directions

There are some limitations in this study. First, different concentrations of SA were not designed in the animal model to observe the protective effect of SA on myocardial infarction. Second, the effect of SA on macrophage polarization at different stages after myocardial infarction was not observed. The molecular mechanism by which SA activates PPAR needs to be further studied. Third, we found that SA can activate PPARγ mRNA and protein expression in both cell and animal experiments. However, the mechanism of SA on PPAR should be further studied. This may be a valuable future study direction. In the end, M1 macrophages metabolize arginine *via* nitric oxide synthase (NOS) to increase NO release, while M2 macrophages metabolize it *via* arginase I to synthesize ornithine and urea. We found that the expression of iNOS increased significantly after MI, and significantly decreased in the MI+SA group. However, we did not evaluate the NO release. It is really interesting to evaluate NO release in the future research direction. Whether SA can prevent ventricular arrhythmia after MI is another future research direction. This is the limitation of this study.

## Data Availability Statement

The original contributions presented in the study are included in the article/supplementary material, further inquiries can be directed to the corresponding authors.

## Ethics Statement

The animal study was reviewed and approved by Animal Ethics Committee of Wuhan Third Hospital, China.

## Author Contributions

MY, JX, and QZh designed this study. MY, JX, QZo, XW, KH, and QZh performed the experiments. MY, JX, and QZo collected and analyzed the data. MY drafted the first manuscript. JX, QZo, and XW revised the manuscript. KH and QZh determined the final manuscript. All authors contributed to the study and approved the final manuscript.

## Funding

This work was supported by the National Natural Science Foundation of China (Grant Numbers 81970082 and 81970277).

## Conflict of Interest

The authors declare that the research was conducted in the absence of any commercial or financial relationships that could be construed as a potential conflict of interest.

## Publisher's Note

All claims expressed in this article are solely those of the authors and do not necessarily represent those of their affiliated organizations, or those of the publisher, the editors and the reviewers. Any product that may be evaluated in this article, or claim that may be made by its manufacturer, is not guaranteed or endorsed by the publisher.
